# Is It Time to Abandon the Absolute Contraindication to FNAC in Pulmonary Hydatid Cysts? A Case Report and Literature Review

**DOI:** 10.1002/rcr2.70610

**Published:** 2026-05-18

**Authors:** Zahra Sadin, Manouchehr Aghajanzadeh, Mohammadreza Sadin, Maryam Sadin, Arsalan Dadashi, Naghi Ramezani

**Affiliations:** ^1^ Department of Thoracic Surgery Arya Private Hospital Rasht Iran; ^2^ Department of Pulmonology Arya Private Hospital Rasht Iran; ^3^ Faculty of Veterinary Medicine Lorestan University Khorramabad Iran

**Keywords:** anaphylaxis, diagnostic imaging, *Echinococcus granulosus*, fine‐needle aspiration cytology, pulmonary hydatid cyst

## Abstract

Pulmonary hydatid cysts commonly mimic other pathologies on imaging, creating diagnostic challenges. Fine‐needle aspiration cytology (FNAC) has historically been avoided because of concerns about anaphylactic reactions, despite evidence suggesting acceptable safety profiles. A 42‐year‐old female presented with a 2‐year history of intermittent respiratory symptoms. Chest CT revealed an irregular pulmonary opacity in the right lower lobe with central fluid attenuation raising suspicion for lung abscess or malignancy. Laboratory investigations showed elevated inflammatory markers. CT‐guided FNAC was performed without complications. It showed cytological features of a pulmonary hydatid cyst. The patient underwent successful posterolateral thoracotomy with wedge resection, and histopathological examination confirmed the diagnosis. Review of published literature shows that FNAC in hydatid disease, if performed with appropriate precautions, has minimal risk. The incidence of lethal anaphylaxis is extremely low (0.03%), with reversible allergic reactions in 1.7% of procedures. Multiple case series report successful diagnostic outcomes when FNAC is performed by experienced operators under image guidance and availability of anesthesiological support. This technique should not be considered absolutely contraindicated but a valuable tool when used cautiously.

## Graphical abstract

This report challenges the historical belief that fine‐needle aspiration (FNAC) is absolutely contraindicated in suspected pulmonary hydatid cysts. By a case of successful diagnosis without anaphylaxis and a systematic review of the literature, we show that with modern image guidance and precautions, the risk may be lower than traditionally thought. It suggests a potential paradigm shift in clinical practice.

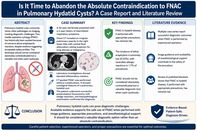

## 1 Introduction

Hydatid disease is a significant public health concern in endemic regions, where sheep farming and close contact with dogs can transmit parasites [1]. While the liver is the most commonly affected organ (70%), the lung is the second most frequent site of involvement. It occurs more commonly in younger people [2].

The diagnosis of pulmonary hydatid disease is based on clinical presentation, epidemiological factors, imaging findings and serological testing. However, these methods have some limitations. Radiological features can mimic other pathologies, including lung abscesses, necrotic malignancies and tuberculous lesions. Especially when faced with a complicated case that rupture, infection, or atypical morphology can mask characteristic findings [3, 4]. Even advanced imaging modalities such as PET‐CT can show false‐positive uptake in metabolically active hydatid cysts. Such results can lead to misdiagnosis of the lung hydatid disease as lung cancer in high‐risk populations [5]. Serological tests show reduced sensitivity in pulmonary hydatidosis compared to hepatic, as false‐negative results are problematic [6, 7].

Fine‐needle aspiration cytology (FNAC) can be a valuable diagnostic tool in selected cases, where non‐invasive methods are unable to provide a definitive diagnosis. FNAC for hydatid cyst has historically been a source of significant clinical concern, because the procedure can cause anaphylactic reactions and also secondary dissemination [8]. Recent studies, however, confirmed favourable outcomes when FNAC is performed with appropriate precautions [9].

This case report presents a patient with pulmonary hydatid cyst, that FNAC played an important diagnostic role, and then followed by surgical treatment. Through systematic review of the literature, we examine the current evidence of the safety, efficacy and appropriate indications for FNAC in pulmonary hydatid disease. It provides an evidence‐based perspective for this diagnostic technique.

## 2 Case Report

A 42‐year‐old female patient presented with a 2‐year history of intermittent respiratory symptoms, including productive cough, dyspnoea, right‐sided chest pain and sputum. Two weeks prior to admission, she developed fever and generalised body aches in addition to her baseline respiratory symptoms. She was initially hospitalised and received medical treatment for 5 days. Her physicians ordered an isolation for 12 days (with assumption of TB); after that, her acute symptoms resolved. Later her respiratory symptoms reoccurred, including cough, dyspnoea, fatigue and sputum. A pulmonologist prescribed her antibiotics and corticosteroids for 7 days. This provided temporary symptomatic improvement. Despite this intervention, her respiratory symptoms persisted and eventually prompted the current presentation.

Physical examination revealed mild respiratory distress with dyspnoea on exertion. Breath sounds were normal bilaterally on auscultation. Vital signs were notable for blood pressure of 127/80 mmHg and pulse rate of 96 beats per minute. Laboratory investigations demonstrated elevated inflammatory markers: erythrocyte sedimentation rate of 45 mm/h (reference range: 0–20 mm/h), C‐reactive protein > 8 mg/dL (reference: < 0.5 mg/dL), and leukocytosis with white blood cell count of 14 × 10^9^/L (reference: 4–11 × 10^9^/L). Additional findings included mild anaemia with haemoglobin of 11 g/dL. Serology test for echinococcosis was negative.

**FIGURE 1:**
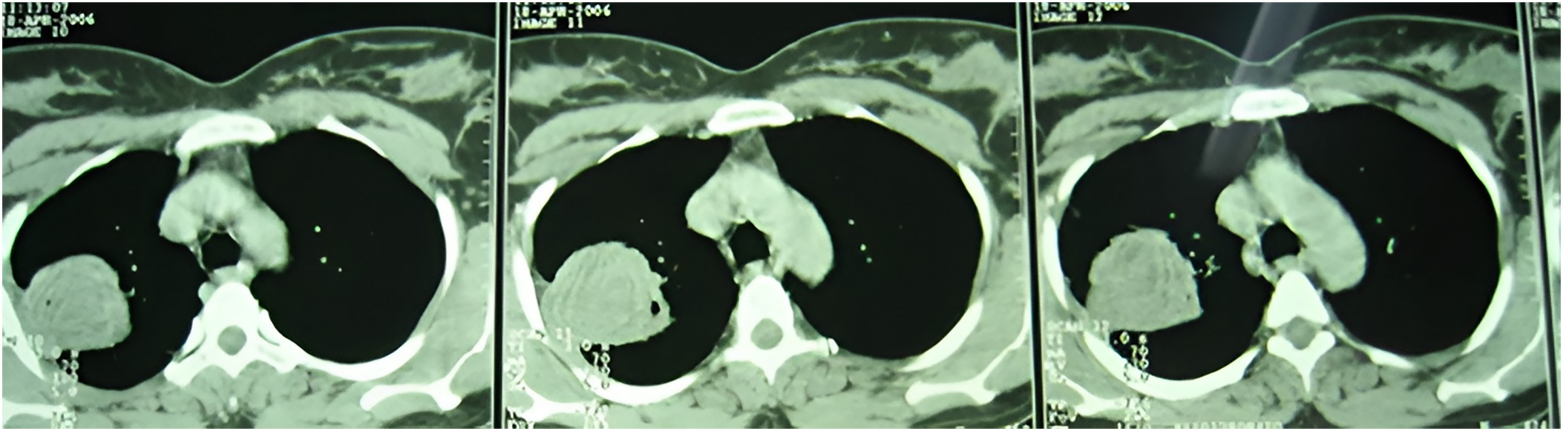
Axial non‐enhanced CT (pulmonary window): A large pulmonary opacity at the right lower lobes which is associated with subtle surrounding pulmonary inflammatory change on the form of surrounding ground glass opacity. Coronal CT image showed pulmonary lesion and central fluid attenuation. The fine needle aspiration needle tip is noted within the presumed wall of the cavitary lesion (not centre).

Despite medical management, her symptoms persisted. Contrast‐enhanced spiral computed tomography (CT) of the chest was performed. It revealed a well‐defined irregular pulmonary opacity in the right lower lobe with central fluid attenuation (Figure 1). The differential diagnosis at this stage included lung abscess versus pulmonary mass.

Figure 1 shows an axial non‐enhanced CT (pulmonary window). There is a large pulmonary opacity in the right lower lobe with surrounding pulmonary inflammatory changes as ground‐glass opacities. Coronal CT reconstruction showed the lesion with apparent central fluid attenuation. The fine‐needle aspiration (FNA) needle tip is visible in the presumed wall of the cavitary lesion.

**FIGURE 2:**
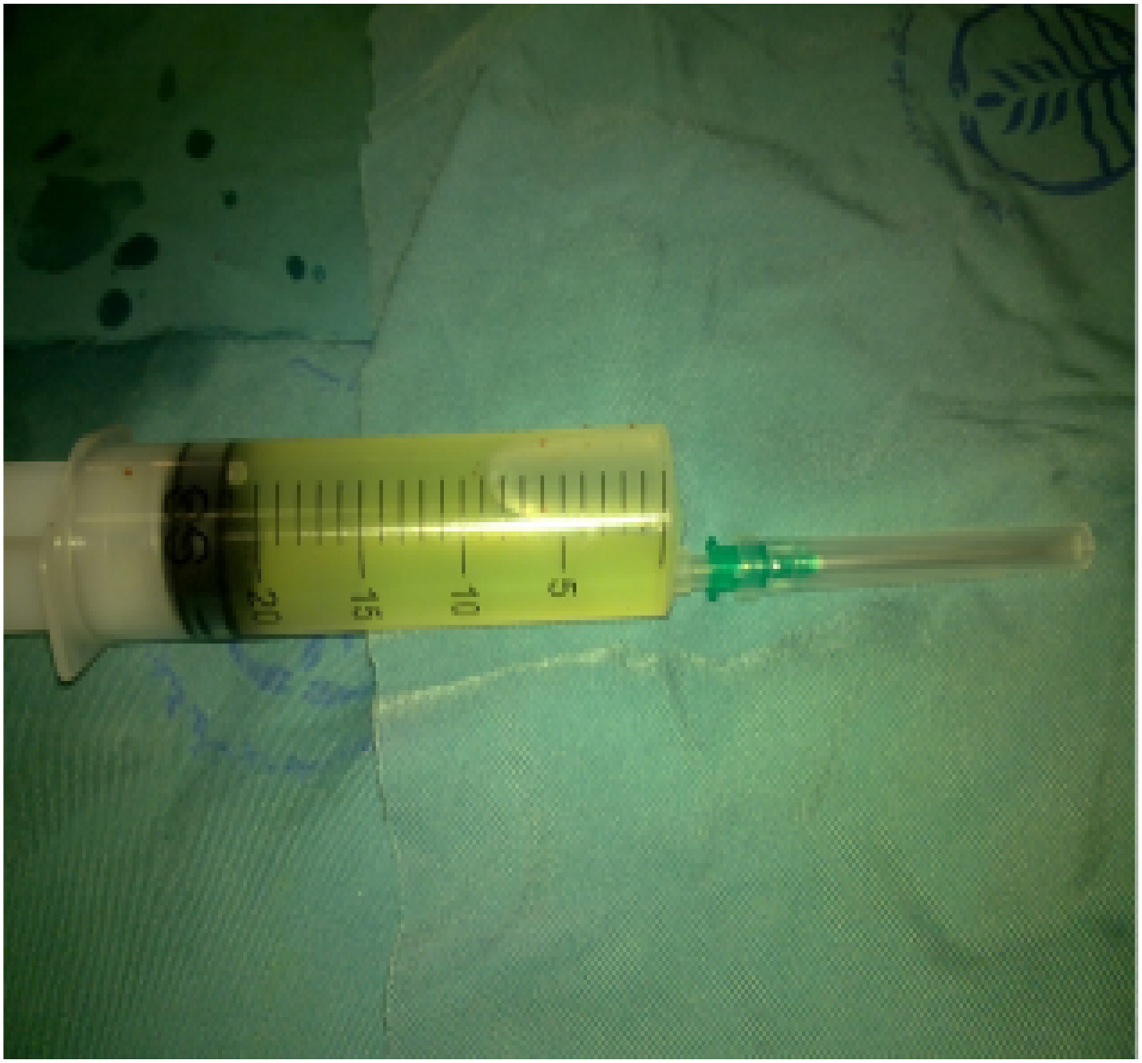
The aspirated material. Histopathologically confirmed pulmonary hydatid cyst.

**FIGURE 3:**
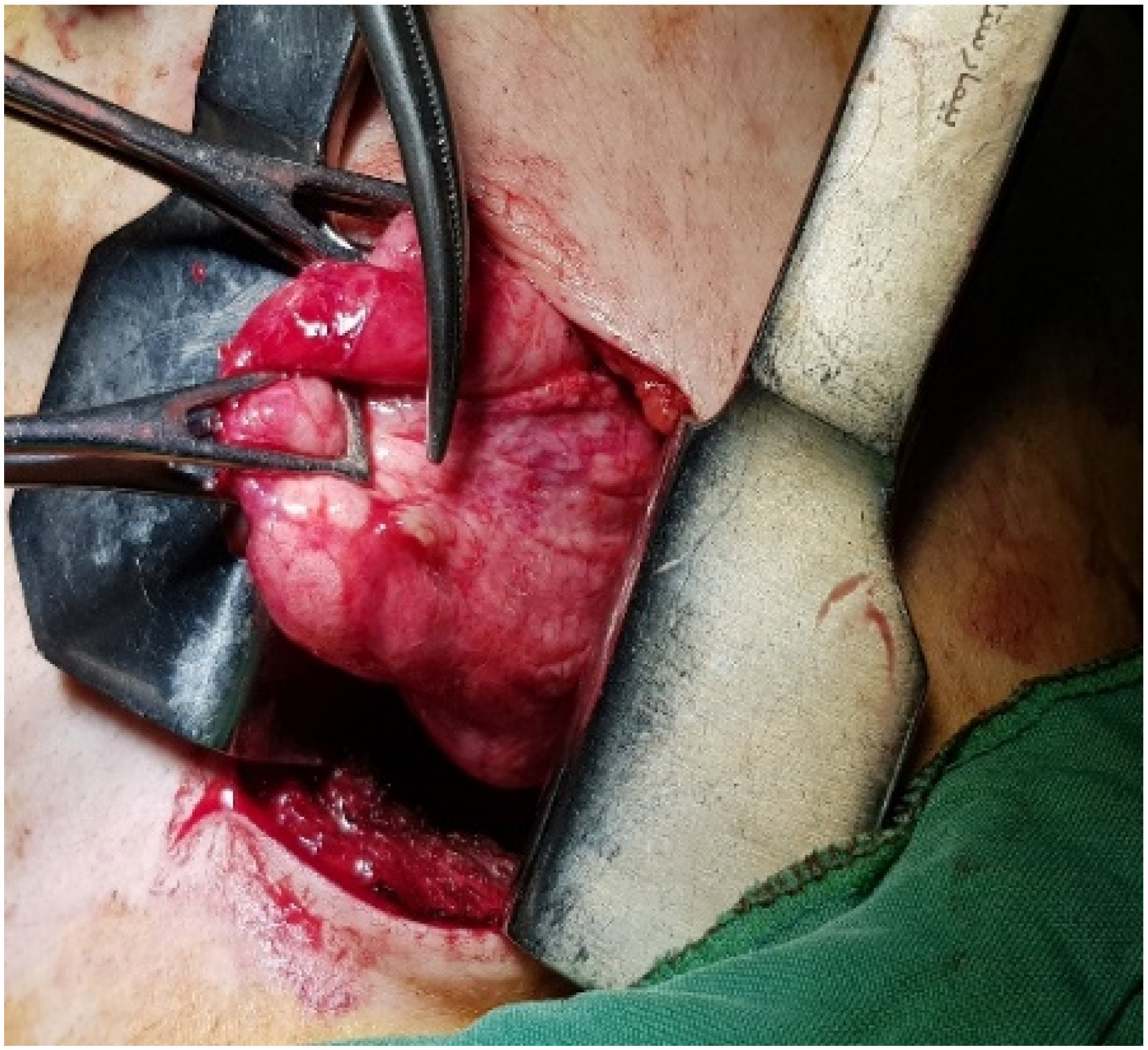
The cyst in the operation room.

**FIGURE 4:**
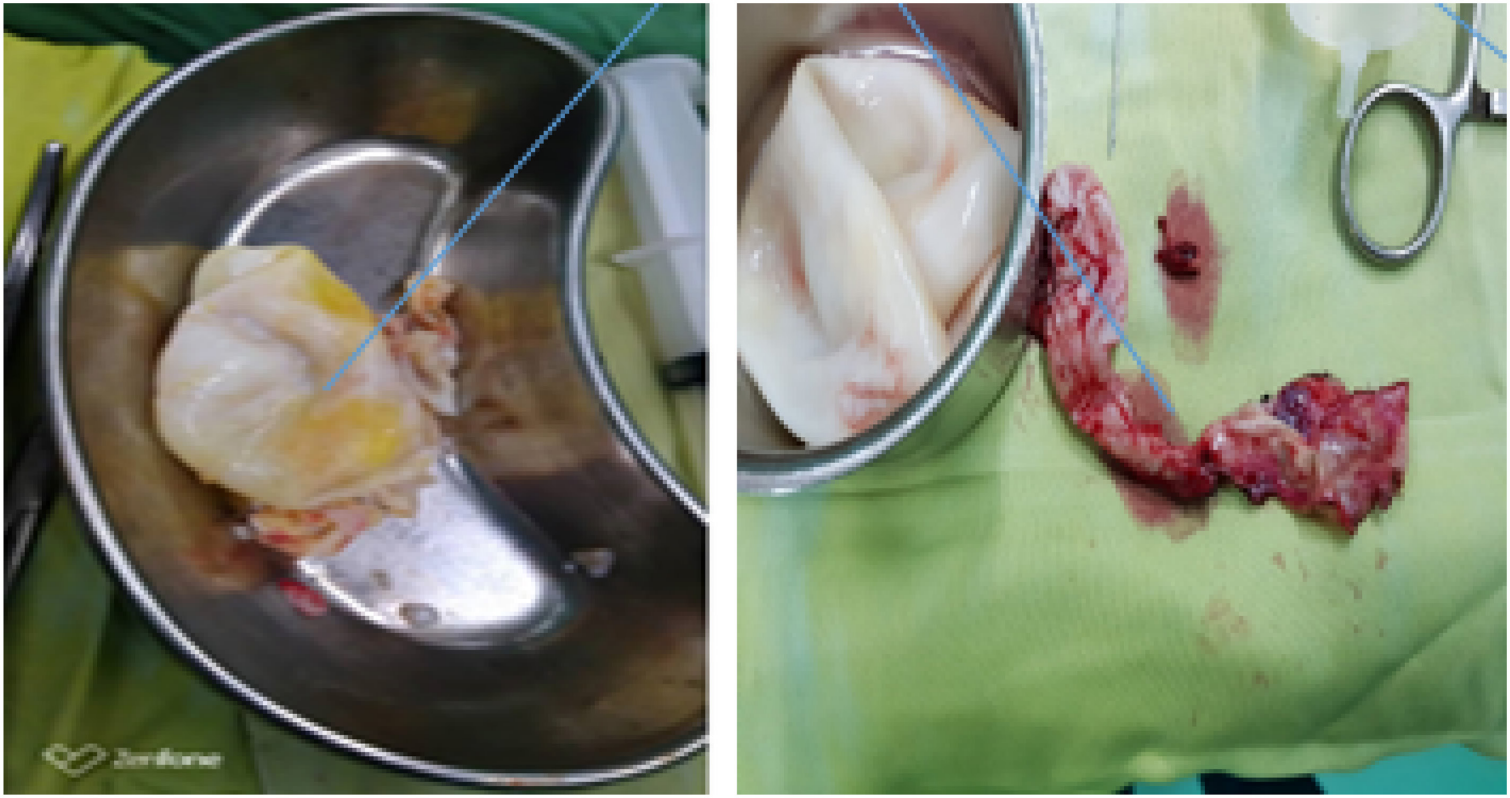
The excised cyst.

**FIGURE 5:**
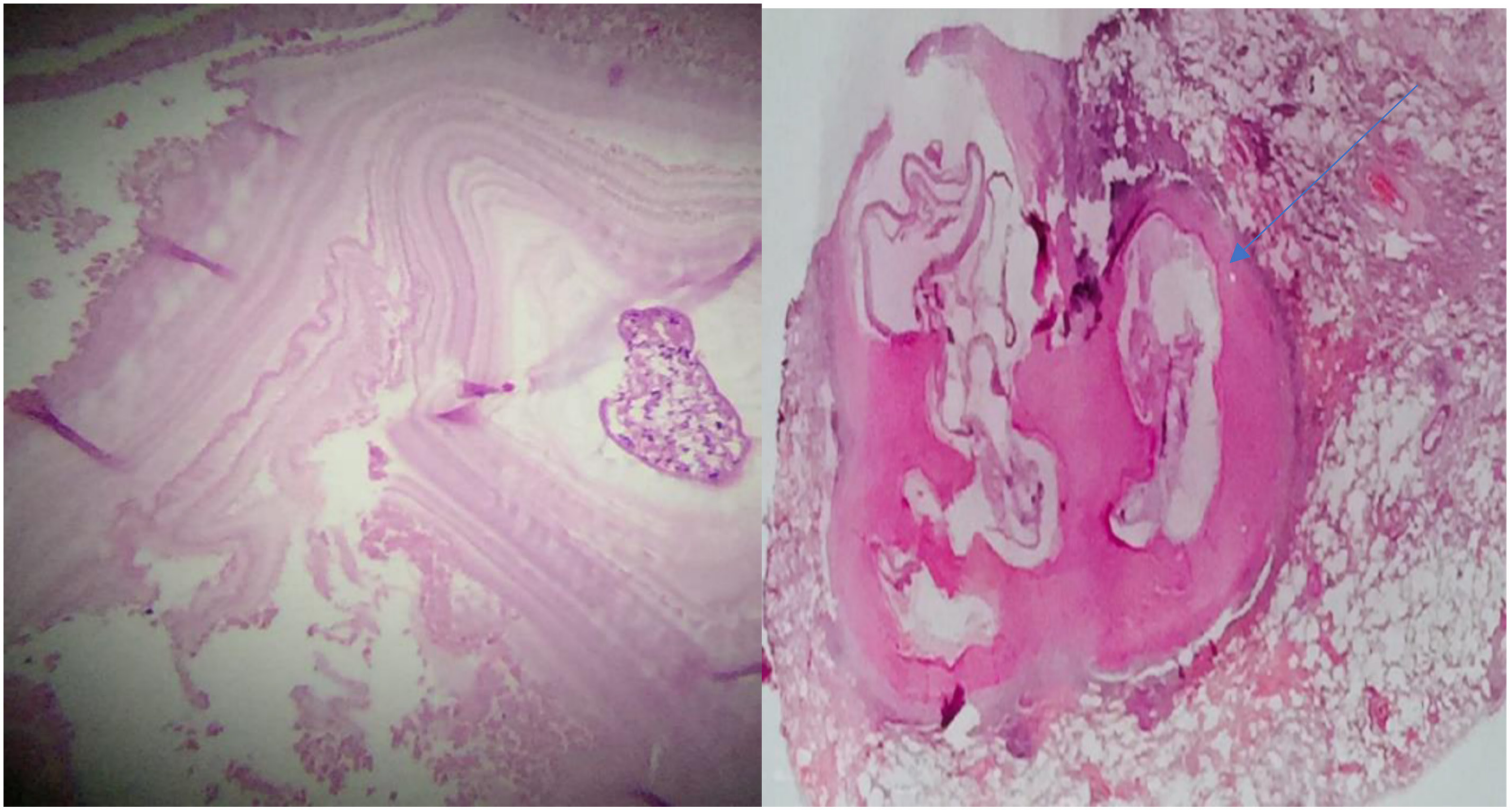
The aspirated material in histopathologic evaluation revealed cellular eosinophilic material containing a laminated membrane (arrow) of hydatid cyst with inflammatory cell infiltrate. It also shows inflamed pulmonary tissue with a hydatid cyst composed of a laminated membrane.

The patient underwent CT‐guided FNA biopsy (FNAB) of the right‐sided lesion in the prone position. It targeted an area of pleural adhesion between the lesion and chest wall. The aspirated material was sent for cytopathological evaluation, which suggested the diagnosis of pulmonary hydatid cyst (Figures 2, 3, 4). Then, the patient underwent posterolateral thoracotomy with wedge resection and complete excision of the cystic lesion. Histopathological examination of the surgical specimen confirmed the diagnosis of pulmonary hydatid cyst (Figure 5).

The postoperative course was uneventful without complications. The patient experienced significant symptomatic improvement and was discharged in stable condition with appropriate antiparasitic therapy and follow‐up arrangements.

We conducted a comprehensive literature search to identify all relevant studies across PubMed/MEDLINE, Scopus, Web of Science and Google Scholar (inception through February 2026). Original studies of FNAC in pulmonary hydatid disease with English language were eligible regardless of their design.

The initial database search identified 150 records. After removal of duplicates and screening of titles and abstracts, 85 full‐text articles were retrieved for detailed review. Following full‐text assessment, 45 studies met the inclusion criteria and were included in the final synthesis. These comprised 28 case reports, 10 case series, 4 observational studies and 3 systematic reviews. Studies were distributed across multiple geographic regions, including endemic areas in the Mediterranean, Middle East, South Asia and South America, as well as non‐endemic regions in North America, Europe and East Asia.

The diagnostic performance of FNAC in pulmonary hydatid disease can be enhanced by identifying the cytological features in the aspirated material. Pathognomonic findings include the visualisation of hooklets and protoscolices, while laminated membrane fragments and daughter cyst elements are highly suggestive but not exclusively diagnostic (Table 1). Agarwal et al. comprehensively characterised the cytological findings of aspirated hydatid fluid. It confirms the morphological basis for cytological diagnosis in this disease [10]. Paksoy et al. showed the clinical utility of FNAC in a case that pulmonary hydatid disease was presenting as a solid nodule and mimicked malignancy. FNAC provided the definitive diagnosis that imaging alone could not establish [11] (Table 2).

**TABLE 1:** FNAC cytological findings.

Cytological feature	Frequency	Diagnostic value
Laminated membrane	Common	Highly suggestive
Hooklets	Variable	Pathognomonic
Protoscolices	Rare in old cysts	Pathognomonic
Daughter cysts	Moderate	Highly suggestive
Inflammatory cells	Common	Nonspecific

**TABLE 2:** Major studies on FNAC diagnostic performance.

Study	Year	Location	*N*	Design	Findings	Complications
Summary of case series on FNAC in pulmonary hydatid cysts						
von Sinner et al.	1995	Saudi Arabia	31	Retrospective	81% no complications; Diagnostic in 23% initially	5 minor allergic reactions, 1 severe hypotension
Das et al.	1995	India	8	Case series	FNAC successful under US guidance	None
Summary of case reports on FNAC in pulmonary hydatid cysts						
Paksoy et al.	2012	Turkey	1	Case report	Mimicked malignancy; diagnosed by FNAC	None
Abdullah et al.	2024	Iraq	1	Case report	Unintentional FNAC; confirmed hydatid	None

The most comprehensively studied aspect of FNAC in hydatid disease is its anaphylaxis risk [6]. These findings are summarised in Table 4. Multiple case reports published between 2009 and 2025 confirmed these findings. Lethal anaphylaxis is still a rare occurrence and is more common in cases of uncontrolled cyst rupture than in planned percutaneous procedures (Table 3). The full spectrum of FNAC‐associated complications, including severe anaphylaxis (0.03%–1%), mild allergic reactions (1.7%–19%), pneumothorax (< 5%), cyst spillage and secondary echinococcosis, is detailed in Table 4.

**TABLE 3:** Anaphylaxis risk assessment.

Study	Year	Sample size	Lethal anaphylaxis	Reversible reactions	Rate
Neumayr et al. (systematic review)	2011	~6000 procedures	0.03%	1.7%	Very low overall risk
von Sinner et al.	1995	31 patients	0%	19%	Minor reactions only
Multiple case reports	2009–2025	Variable	Rare	Occasional	Risk higher with cyst rupture

**TABLE 4:** Complications of FNAC in hydatid cysts.

Complication	Frequency	Severity	Management
Anaphylaxis (severe)	0.03%–1%	Life‐threatening	Immediate resuscitation
Allergic reactions (mild)	1.7%–19%	Minor	Antihistamines, observation
Pneumothorax	< 5%	Moderate	Usually, self‐limited
Cyst spillage	Rare	Variable	Prevention critical
Secondary echinococcosis	Very rare	Serious	Long‐term follow‐up

Across the included studies, CT guidance was the most commonly employed imaging modality for FNAC in pulmonary hydatid disease. It offers good lesion characterisation, real‐time needle visualisation and the ability to plan needle trajectories that minimize the risk of free cyst rupture. Ultrasound guidance was used in several series, particularly for peripheral lesions, and Das et al. showed successful FNAC under ultrasound guidance in 8 patients without complications [12] (Table 2). The consensus across studies preferred targeting sites of pleural adhesion between the cyst and chest wall. This approach is thought to reduce the risk of intrapleural cyst content spillage. The role of operator experience was consistently emphasised as a critical determinant of procedural safety and diagnostic yield [7, 9].

The performance characteristics of imaging modalities are summarised in Table 5. CT signs (water‐lily sign, daughter cysts, calcification; loss of features in complicated/ruptured cysts; false‐positive PET‐CT), MRI (T2 laminated membrane, daughter cyst signal, use as second‐line), ultrasound (double‐layer wall sign, 100% specificity in selected series, pediatric use) and chest X‐ray (crescent sign, limited specificity).

**TABLE 5:** Imaging Modalities Performance.

Modality	Sensitivity	Specificity	Key findings
CT	80.9%	High	Best for complicated cysts, calcification
MRI	High	High	Superior for cyst contents, membrane visualisation
Ultrasound	Variable	Variable	‘Wall sign’ 100% specific; good for paediatrics
Chest X‐ray	Low‐Moderate	Low	Initial screening only

CT has the highest overall utility, with a sensitivity of 80.9% and superior performance in complicated cysts and calcified lesions [3]. MRI offered high sensitivity and specificity with particular advantages for cyst content characterisation and membrane visualisation, but its role is adjunctive to CT in most clinical settings. Ultrasound has variable performance but has 100% specificity with the wall sign in selected series. It is particularly useful in paediatric populations. Chest radiography serves as an initial screening tool only, with limited sensitivity and specificity for definitive diagnosis.

The serological diagnostic performance data extracted from included studies are summarised in Table 6. ELISA IgG demonstrated overall sensitivity of 80%–85% but pulmonary‐specific sensitivity of only 40%–84%, with specificity of 87%–100% [6]. IHA showed overall sensitivity of 80%–84% with pulmonary sensitivity of 60%–74% and specificity of 73%–87%. Immunoblotting has the highest specificity at 99% but variable sensitivity.

**TABLE 6:** Serological tests performance.

Test	Sensitivity (overall)	Sensitivity (pulmonary)	Specificity	Notes
IHA	80%–84%	60%–74%	73%–87%	Lower sensitivity in lung cysts
ELISA IgG	80%–85%	40%–84%	87%–100%	Most commonly used
Immunoblotting	81%	Variable	99%	Highest specificity
IEP	62%	Variable	99%	Lower sensitivity
Combined tests	94.7%	Higher	Variable	Recommended approach

Evidence‐based prevention for complications during FNAC in hydatid disease is summarised in Table 7. High‐evidence recommendations include the use of an experienced operator, mandatory image guidance using CT or ultrasound, immediate anaesthesia standby and availability of resuscitation equipment. Informed consent which documents the specific risks of anaphylaxis, spillage and secondary dissemination is mandatory. Moderate‐evidence recommendations include preoperative antihistamine administration in high‐risk cases and albendazole. Although albendazole is not agreed upon by some authors.

**TABLE 7:** Prevention strategies for FNAC complications.

Strategy	Evidence level	Recommendation
Experienced operator	High	Strongly recommended
Image guidance (CT/US)	High	Mandatory
Preoperative antihistamines	Moderate	Consider in high‐risk cases
Anaesthesia standby	High	Essential
Resuscitation equipment	High	Must be available
Informed consent	High	Mandatory
Albendazole coverage	Moderate	Recommended by some

Abbreviations: CT = computed tomography; ELISA = enzyme‐linked immunosorbent assay; IEP = immunoelectrophoresis; IHA = indirect hemagglutination assay; *N* = number of patients/procedures; US = ultrasound.

The largest contributions originated from the Middle East (15 studies) and Mediterranean regions (12 studies). It reflects the high endemic burden in these areas. South Asia contributed 8 studies, while East Asia, Europe and North America contributed fewer studies.

## 3 Discussion

This case shows the diagnostic challenge of pulmonary hydatid cysts and highlights the role of FNAC when traditional diagnostic approaches are inconclusive. Our patient received empirical antibiotics and corticosteroids and was evaluated for possible malignancy over a two‐year symptomatic course. Then tissue diagnosis was established by CT‐guided fine‐FNAC.

While prior reports have described similar diagnostic delays [4, 10], this case is special because the CT findings (irregular margins, central fluid attenuation and surrounding ground‐glass opacity) met the imaging criteria for both pyogenic lung abscess and necrotic malignancy. The serological results did not resolve this unclarity. These diagnostic uncertainties caused limitations in conventional approaches, and tissue sampling became the clinically appropriate next step. The diagnostic challenge in this case shows a common clinical scenario.

What distinguishes this case from prior reports is the systematic integration of that diagnostic decision. The central argument is not that FNAC should be performed routinely in suspected hydatid disease (it should not), but instead that the lethal anaphylaxis rate is 0.03% and those events were hepatic, not pulmonary. The diagnostic alternatives can fail in pulmonary disease (serology sensitivity as low as 40%) and every published pulmonary case series reported success without fatal reactions. The absolute prohibition itself is harmful, because it forces empirical thoracotomy [6], and this restriction may itself cause delay in definitive diagnosis.

In this case, CT is the main choice of initial evaluation. When pathognomonic features such as the water‐lily sign, daughter cysts, or membrane detachment are present, CT can achieve a sensitivity of 80.9%. However, these findings are absent in uncomplicated cysts or in complicated cases with atypical morphology [3]. Furthermore, even advanced imaging modalities are not immune to misclassification: false‐positive PET‐CT uptake has been documented in metabolically active hydatid cysts. It can mimic lung cancer in high‐risk populations. It confirms that this disease has potential for misdiagnosis. In such conditions, FNAC should be considered [3, 4].

Serological testing has a documented limitation in pulmonary hydatidosis. Its sensitivity is lower than in hepatic disease, ranging from 40%–84% for ELISA IgG and 60%–74% for indirect hemagglutination assay (IHA) in pulmonary cases, compared with 80%–85% and 80%–84%, respectively for hepatic disease. It means that a negative serological result cannot reliably exclude the diagnosis [5]. In our patient, the combination of atypical imaging and serological uncertainty created a diagnostic gap that could not be bridged without tissue sampling. A combined testing approach using both ELISA and IHA can be recommended to enhance sensitivity, but it may not be universally available [5].

FNAC is the most valuable tool when imaging and serology are non‐diagnostic, and when tissue diagnosis will change patient management [9]. In our case, FNAC showed cytological identification of laminated membrane fragments. While this finding is highly suggestive, it is less pathognomonic than visualisation of hooklets or protoscolices [8]. The cytological findings of aspirated hydatid fluid, including laminated membrane material, scolices, hooklets and daughter cyst elements, are well characterised [8].

The traditional contraindication to FNAC in hydatid disease originated from early concerns about anaphylactic reactions and secondary dissemination after cyst puncture [9]. Most of the documented anaphylactic reactions and secondary dissemination occurred in hepatic hydatid cyst aspiration. They were often without modern image guidance. Published studies about pulmonary FNAC series have recorded no life‐threatening anaphylaxis (only minor reactions). CT‐guided pulmonary FNAC targets the cyst wall at pleural adhesion. It is fundamentally different from hepatic aspiration. These concerns were clinically reasonable in the absence of systematic data, and they have historically created significant anxiety among physicians [9]. However, the publication of Neumayr et al.'s systematic review, which evaluated 6000 percutaneous procedures for cystic echinococcosis, changed the situation [8]. Their analysis found a lethal anaphylaxis rate of only 0.03% and reversible allergic reactions in only 1.7% of procedures. It shows that concerns about this complication have been exaggerated [8]. A case series of von Sinner et al. reported no life‐threatening reactions in 31 patients undergoing FNA biopsy of hydatid cysts and only 5 minor allergic reactions and 1 episode of severe but reversible hypotension, confirming this conclusion [9]. Similarly, Abdullah et al. described a case of unintentional FNAC of a pulmonary lesion, which initially was suspected to be malignancy. It confirmed hydatid disease without anaphylaxis and led directly to successful surgical treatment [13].

A 0.03% lethal anaphylaxis rate, while low, can be a preventable death in a disease with excellent surgical outcomes [6]. The appropriate clinical presumption is not that FNAC is unconditionally safe, but that it is safe with precautions, specifically, performance by an experienced operator, CT guidance for accurate needle placement, targeted aspiration at sites of pleural adhesion to minimize spillage risk, and immediate availability of anesthesiological support and resuscitation [7, 9]. Our case shows that this protocol is achievable and clinically effective.

This case report and literature review has some limitations.

This literature review is limited by the quality of the existing studies. Of 45 included studies, 28 were case reports, and the observational studies have limited sample sizes. No randomized controlled trials exist due to the rare nature of the disease and the ethical constraints on randomizing patients to biopsy of a suspected hydatid cyst. The reported complication rates should therefore be interpreted with caution: the true rates are probably underreported because of publication bias that favours simpler procedures, and they reflect heterogeneous technical protocols that prevent direct statistical analysis [6].

Geographic variation in *Echinococcus granulosus* strains may affect cyst characteristics, membrane integrity and antigen expression [1]. Most of included studies originated from Mediterranean and Middle Eastern endemic regions, and their findings may not be fully generalizable to non‐endemic settings. As clinical experience with the disease is more limited there [1].

These limitations confirm the need for prospective multicentre studies with standardized protocols to advance the evidence base for FNAC in pulmonary hydatid disease.

We propose a stepwise approach to the diagnostic evaluation of suspected pulmonary hydatid cysts. The framework limits FNAC to cases with specific diagnostic value [6, 7, 9].

The initial evaluation should include a thorough clinical and epidemiological assessment. Also, clinicians should pay special attention to residence in or travel to endemic regions, occupational exposure to livestock and contact with dogs [1]. Chest CT with contrast should be performed in all patients to characterise lesion morphology and assess for pathognomonic imaging features [3]. Serological testing using both ELISA and IHA should be performed concurrently. But clinicians should consider that a negative result does not exclude pulmonary disease [5].

When imaging findings are characteristic and serology is positive, albendazole should be started and then surgical planning [2, 13]. When diagnostic uncertainty persists, either because imaging is atypical or serology is negative, candidacy for CT‐guided FNAC should be evaluated [6, 7]. FNAC is appropriate when an experienced operator is available, when immediate anesthesiological support and resuscitation equipment are on hand, when the lesion is accessible with the needle and when tissue diagnosis will directly change clinical management [7, 9]. Following confirmed cytological diagnosis of hydatid disease, surgical planning proceeds with antiparasitic coverage, and long‐term imaging surveillance is mandatory [2, 9].

Clinicians in endemic regions should have a high index of suspicion for pulmonary hydatid disease in any cavitary or cystic pulmonary lesion, even when imaging suggests an alternative diagnosis [1, 3]. The diagnostic yield of FNAC is sufficient to guide management when characteristic cytological features are identified [8].

Several research priorities are in this review. First, the prospective documentation of FNAC outcomes in pulmonary hydatid disease with standardized complication (like anaphylaxis grading, haematological parameters, imaging guidance modality and operator experience). It would strengthen an evidence base that relies on retrospective case series [6]. Second, the further investigation of cytological diagnostic criteria would help standardize the interpretation of FNAC specimens in degenerated or older cysts such as when we identify a laminated membrane in the absence of hooklets or protoscolices [8]. Third, the evaluation of combined diagnostic approaches (advanced serological testing with targeted FNAC) may create a better strategy for tissue sampling and minimize both diagnostic delay and procedural risk [5, 9].

Further prospective studies with standardized protocols can modify indications and improve safety measures of this diagnostic technique.

In conclusion, pulmonary hydatid cyst is a diagnostic challenge that imaging and serology alone cannot always resolve [3, 6]. This case shows that CT‐guided FNAC can provide a definitive diagnosis in carefully selected patients without precipitating anaphylaxis or other serious complications. It can be safe if performed by an experienced operator with full anesthesiological support and resuscitation readiness [7, 10]. The evidence in the literature does not support an absolute contraindication to FNAC in hydatid disease; it supports instead a protocol‐driven approach. The procedure should be reserved for cases when the diagnostic yield outweighs the procedural risk [7, 9]. A multidisciplinary approach combining imaging, serology, selective FNAC and surgical expertise is the most evidence‐based approach for managing these complex cases [2, 9].

## Author Contributions

**Zahra Sadin:** conceptualisation; methodology; writing – original draft; writing – review and editing. **Manouchehr Aghajanzadeh:** conceptualisation; methodology; writing – original draft; writing – review and editing. **Mohammadreza Sadin:** data curation; investigation; resources. **Maryam Sadin:** formal analysis; visualisation. **Arsalan Dadashi:** validation; supervision. **Naghi Ramezani:** project administration; supervision; writing – review and editing. All authors reviewed and approved the final version of the manuscript and agree to be accountable for all aspects of the work.

## Funding

The authors have nothing to report.

## Consent

The authors declare that written informed consent was obtained for the publication of this manuscript and accompanying images using the form provided by the Journal.

## Conflicts of Interest

The authors declare no conflicts of interest.

## Data Availability

All data supporting the findings of this study are contained within the manuscript.

## References

[1] J. Eckert and P. Deplazes , “Biological, Epidemiological, and Clinical Aspects of Echinococcosis, a Zoonosis of Increasing Concern,” Clinical Microbiology Reviews 17, no. 1 (2004): 107–135. 14726458 10.1128/CMR.17.1.107-135.2004 PMC321468

[2] R. Bagheri , S. Z. Haghi , M. Amini , A. S. Fattahi , and S. Noorshafiee , “Pulmonary Hydatid Cyst: Analysis of 1024 Cases,” General Thoracic and Cardiovascular Surgery 59, no. 2 (2011): 105–109. 21308436 10.1007/s11748-010-0690-z

[3] M. K. Garg , M. Sharma , A. Gulati , et al., “Imaging in Pulmonary Hydatid Cysts,” World Journal of Radiology 8, no. 6 (2016): 581–587. 27358685 10.4329/wjr.v8.i6.581 PMC4919757

[4] M. Aghajanzadeh , A. Tangestaninejad , S. A. F. Mosavi , et al., “Presentation, Diagnosis, Management and Outcome of Complicated Hydatid Cyst of the Lung,” GSC Advanced Research and Reviews 18, no. 2 (2024): 362–373.

[5] M. A. Zahra Sadin , M. Sadin , and A. A. Foumani , “Can a Solitary Pulmonary Nodule With Positive PET Scan in a Heavy Smoker be Something Other Than Lung Cancer? A Case Report of Pulmonary Hydatid Cyst and Literature Review,” Clinical Case Reports 14, no. 4 (2026): e72175. 42022007 10.1002/ccr3.72175 PMC13095870

[6] F. N. Eris , C. Akisu , and U. Aksoy , “Evaluation of Two ELISA and Two Indirect Hemagglutination Tests for Serodiagnosis of Pulmonary Hydatid Disease,” Korean Journal of Parasitology 47, no. 4 (2009): 427–429. 19967097 10.3347/kjp.2009.47.4.427 PMC2788728

[7] H. Zait and B. Hamrioui , “Human Cystic Echinococcosis: Serological Diagnosis by Indirect Hemagglutination Test, Enzyme‐Linked Immunosorbent Assay, Immunoelectrophoresis, and Immunoblotting in Surgically Confirmed Patients Versus Cases Diagnosed by Imaging Techniques,” Médecine et Maladies Infectieuses 50, no. 8 (2020): 676–683. 31727467 10.1016/j.medmal.2019.10.001

[8] A. Neumayr , G. Troia , C. de Bernardis , et al., “Justified Concern or Exaggerated Fear: The Risk of Anaphylaxis in Percutaneous Treatment of Cystic Echinococcosis‐a Systematic Literature Review,” PLoS Neglected Tropical Diseases 5, no. 6 (2011): e1154. 21695106 10.1371/journal.pntd.0001154 PMC3114754

[9] W. N. von Sinner , R. Nyman , T. Linjawi , and A. M. Ali , “Fine Needle Aspiration Biopsy of Hydatid Cysts,” Acta Radiologica 36, no. 2 (1995): 168–172. 7710798

[10] P. K. Agarwal , N. Husain , and B. N. Singh , “Cytologic Findings in Aspirated Hydatid Fluid,” Acta Cytologica 33, no. 5 (1989): 652–654. 2781968

[11] N. Paksoy , D. Ozer , and I. Tuneli , “Diagnosis of Pulmonary Hydatid Disease Presenting With Solid Nodule and Mimicking Malignancy by Fine Needle Aspiration Cytology,” CytoJournal 9 (2012): 13. 22615711 10.4103/1742-6413.95832 PMC3352695

[12] D. K. Das , S. Bhambhani , and C. S. Pant , “Ultrasound Guided Fine‐Needle Aspiration Cytology: Diagnosis of Hydatid Disease of the Abdomen and Thorax,” Diagnostic Cytopathology 12, no. 2 (1995): 173–176. 7774501 10.1002/dc.2840120219

[13] A. M. Abdullah , R. J. Rashid , S. H. Tahir , et al., “Diagnosis of a Pulmonary Hydatid Cyst by Fine Needle Aspiration: A Case Report With Literature Review,” Annals of Medicine and Surgery 86, no. 1 (2024): 552–555. 38222674 10.1097/MS9.0000000000001526 PMC10783388

